# The Technology of Bacteria Investigation

**Published:** 1885-09

**Authors:** 


THE TECHNOLOGY OF BACTERIA INVESTIGATION. By Chas. S. Dolley, M. D. 12 mo. pp. 263. Boston: S. E. Cassino & Co. 1885.

Bacteriological studies have, in the last decade, developed remarkably, and the literature relating thereto is beginning to assume great proportions. The volume before us possesses a peculiar value, in that it forms a collection of knowledge hitherto scattered throughout the medical literature of the world, and in most cases inaccessible, especially to an American. The author gives a complete list of the methods which are employed by the most distinguished mycologists in the study of bacteria.

The book is divided into two parts—part first containing *genecal directions;* part second, *special methods for investigating pathogenic bacteria.* Part first is divided into four subdivisions—first, *microscopical preparations*, in which the author gives the habitat of various kinds of bacteria, and the methods whereby the living forms are collected and prepared for microscopical examination. Then follows a list of stainings and general directions for the study of fixed and hardened bacteria. Second, *culture experiments*, where are given the methods of sterilization, an enumeration of various instruments for cultures, and directions for preparing culture media. Third, *vaccination and inoculation experiments.* Under this head is described the manner in which these experiments are performed. Fourth, *biological analysis.* Here a scheme or list of questions is presented which is to be followed out in determining the life-history of these organisms. Part second gives the most approved methods of preparing specimens of many of the pathogenic bacteria, and especially complete are the methods relating to the bacillus tuberculosis.

Part third consists of a formulary in which are embraced many standard formula for culture fluids, reagents, mounting material, etc., necessary in carrying on bacteridian work.

A novel feature, and one which becomes apparent at a glance, is the classification of the bibliography, and its distribution in the body of the book immediately after the subject it represents. The references are unusually full, and this, together with their appropriate arrangement, is of great advantage to the investigator, and adds greatly to the value of the book. The style of the author is clear and concise, and we congratulate him on the successful manner in which he has accomplished a very laborious task. H. W.

